# Long-term self-reported attendance in exercise training or lung choir and status of quality of life following initial pulmonary rehabilitation for COPD

**DOI:** 10.3389/fresc.2024.1447765

**Published:** 2024-09-19

**Authors:** Mette Kaasgaard, Uffe Bodtger, Søren T. Skou, Stephen Clift, Ole Hilberg, Daniel Bech Rasmussen, Anders Løkke

**Affiliations:** ^1^ Pulmonary Research Unit (PLUZ), Department of Medicine, Zealand University Hospital, Naestved, Denmark; ^2^ Department of Regional Health Research, Faculty of Health Sciences, University of Southern Denmark, Odense, Denmark; ^3^ The Research and Implementation Unit PROgrez, Department of Physiotherapy and Occupational Therapy, Næstved-Slagelse-Ringsted Hospitals, Slagelse, Denmark; ^4^ Research Unit for Musculoskeletal Function and Physiotherapy, University of Southern Denmark, Odense, Denmark; ^5^ Sidney De Haan Research Centre for Arts and Health, Canterbury Christ Church University, Canterbury, United Kingdom; ^6^ International Centre for Community Music, York St John University, York, United Kingdom; ^7^ Department of Medicine, Lillebaelt Hospital, Vejle, Denmark

**Keywords:** chronic obstructive pulmonary disease, pulmonary rehabilitation, long-term attendance, quality of life, physical exercise training, group singing, lung choir, completion rate

## Abstract

**Background:**

Both adherence rates to pulmonary rehabilitation (PR) programmes and long-term attendance in exercise training after PR remain a challenge. In our previous randomised controlled trial (RCT), effects were positively associated with a dose-response pattern, regardless of whether PR contained conventional physical exercise training (PExT) or Singing for Lung Health (SLH) as a training modality within a 10 weeks’ PR programme for chronic obstructive pulmonary disease (COPD). However, long-term status of this RCT cohort remains unknown. In this study, we investigated whether current status (=attendance in supervised exercise training or a lung choir and scoring in quality of life (QoL)) was related to initial PR completion, randomisation, or adherence.

**Methods:**

We collected data via telephone, using a researcher-developed questionnaire on current self-reported attendance in supervised exercise training or a lung choir and on perceived benefits of the initial RCT intervention. Additionally, we used COPD-validated questionnaires (primarily: QoL (measure: St George's Respiratory Questionnaire; SGRQ).

**Results:**

In 2023 (i.e., mean/median 4.7 years after initial PR), surviving participants were contacted (*n* = 196; 73% of 270), and 160 (82% of 196) were included. Out of the included participants, 30 (19%) had not completed initial PR. Compared to the initial PR-completers, non-completers reported less current attendance in exercise training or lung choir (24% vs. 46%, *p* = 0.03) but SGRQ scores were comparable. Yet, those who attended exercise training or lung choir at present (*n* = 66/160; 41% out of 160) reported better QoL score than those with no current attendance (SGRQ; Attending: 39.9 ± 15.4; Not attending: 43.1 ± 16.7; *p* = 0.02). Neither having had SLH instead of PExT, nor adherence level during initial PR, was related to current attendance or to QoL scores.

**Conclusion:**

This study indicates that long-term self-reported attendance and current QoL scores are positively related to initial completion of a PR programme. Surprisingly, neither initial PR content (PExT or SLH) nor initial PR adherence was related to long-term outcomes. We suggest that future PR programmes include special attention to those who do not complete PR to support long-term attendance and QoL status.

## Introduction

Pulmonary rehabilitation (PR) is a short-term, multidisciplinary intervention and a cornerstone in care for people with chronic obstructive pulmonary disease (COPD) to enhance physical capacity and quality of life (QoL), to support lifestyle changes, and to prevent long-term sedentary behaviour (1–4). Physical exercise training (PExT) is a key component in PR. Completion rates and adherence levels to PR with PExT, however, remain suboptimal (1, 3, 5, 6) and research investigating evidence of new activities has been requested as supplement or alternative to PExT within a more personalised and motivating PR offer (1–3). Besides a strengthening of future PR offers, a structured maintenance programme appears to be essential to support lifestyle changes and to preserve effects achieved during initial PR, as effects otherwise seem to fade away within the first year after attending a PR programme (3, 7–9). Especially, supervised physical exercise training is suggested to preserve exercise capacity and QoL (3, 7, 8), but an optimal long-term maintenance model has not yet been defined or established (2, 3, 7, 8).

To address the request for novel, potential activities as part of PR, we recently conducted a multicentre RCT (August 2017 to May 2019) within a 10-week community-based PR programme for COPD. In this initial RCT, we compared standard PExT with singing as an alternative training modality, delivered as the disease-specific approach, Singing for Lung Health (SLH) (10–12). The study demonstrated short-term effects on both physical capacity (measured: Six-minute walk test distance) and QoL (measure: St George's Respiratory Questionnaire; SGRQ) in a dose-response pattern, regardless of study arm (10, 12–15). Neither completion rate nor adherence level differed between study arms (12, 16). *Post hoc* analyses indicated that SLH was related to improved dyspnoea control, inspiratory muscle strength, and heart rate response (17).

After the PR programme, study participants in both study arms were encouraged to implement an active lifestyle with continued exercise, although no formal maintenance programme was available in their local communities. As our previous study design did not include structured follow-up assessments, it remains unknown whether the study cohort maintained exercise attendance and QoL status. Moreover, it remains unclear whether there would be any impact on exercise attendance and QoL status of completion status during initial PR (2, 4), of initial participation in SLH as part of PR, or of adherence level during the initial PR programme.

For the present study, we considered long-term “attendance” as either current self-reported attendance in supervised exercise training (=with a trainer present) or in community-based group singing for lung patients (=with a singing teacher/choir conductor present), given that the participants were introduced to these two activities during the initial RCT and given that there was no formal maintenance programme avaliable. To note, group singing for lung patients is termed “lung choir” in Denmark, however, heterogeneously delivered and yet without any standardised training for the singing teachers (18).

## Aims and hypotheses

In the present study, we aimed to conduct a long-term follow-up study in the initial RCT cohort to explore any long-term impact of completion status, randomisation, and adherence level during initial PR.

Specifically, we hypothesised that (1) current self-reported attendance in supervised exercise training or a lung choir, current QoL score (secondarily: dyspnoea score, and symptoms of anxiety and depression score), and perceived benefits of the initial RCT intervention are related to PR completion status during the initial RCT.

Moreover, we hypothesised that (2) current self- reported attendance in supervised exercise training or a lung choir was related to current QoL score (secondarily: dyspnoea score, and symptoms of anxiety and depression score) and to perceived benefits of the initial RCT intervention.

In addition, we hypothesised that current self-reported attendance in supervised exercise training or a lung choir, current QoL score (secondarily: dyspnoea score, and symptoms of anxiety and depression score), and perceived benefits of the initial RCT intervention (3) are not related to initial randomisation (SLH or PExT), and (4) are related to PR adherence level during the initial RCT.

## Methods

### Study design and oversight

Between February and May 2023, we conducted an observational long-term follow-up study in the cohort of our initial RCT, which had been conducted between August 2017 and May 2019 (12)*.* The study was performed in accordance with the Helsinki II Declaration and was approved by the local ethics committee (REG-135-2022). A statistical analysis plan for long-term outcomes was uploaded June 19th 2023 at ClinicalTrials.gov (NCT03280355).

### Participants

We aimed to include the full study cohort of the initial RCT (*n* = 270/29 clusters) (12).

Participants who were still alive were contacted by telephone and informed about the study. Those declining to participate were registered along with those not reached within three attempts.

### Data collection procedure

We collected data on basic characteristics, used a researcher-developed questionnaire, and repeated specific COPD-related, validated questionnaires from the initial RCT. Study participants provided their answers via telephone and data were entered directly into secure web-based database SurveyXact by Ramboll (Rambøll Management Consulting, Aarhus, Denmark) by a research assistant. In June 2023, data were imported into statistical software STATA 18 (StataCorp LLC, Texas, USA), anonymised, cleaned, and prepared before merging 1:1 to observations with the initial, relevant RCT data. Merging and subsequent analysis was initiated July 6th and completed October 6th 2023.

#### Blinding

The research assistant who performed data collection and data entry was blinded to variables of interest, including to randomisation and performance in the initial RCT. For procedures related to blinding in the initial RCT, please see the original RCT report (12).

### Outcomes and measures

#### Basic characteristics

We collected self-reported, basic data about COPD-related medicine consumption, exacerbations, GP visits, and hospitalisations within the last year, together with current smoking status and level of consumption. Moreover, we imported initial RCT data on socio-demographic characteristics, initial randomisation, age, body-mass index (BMI), sex, COPD-specific characteristics including lung function (expressed as forced expiratory volume in 1 s, % of predicted; FEV% predicted), and RCT performance. See Table 1 for details (additional RCT data related to the present study cohort are provided in Supplementary Tables S1 and S3).

**Table 1:** Initial PR completion status related to characteristics, attendacen in exercise training or lung choir, quality of life, symptoms of anxiety and depression, dyspnoea, and perceived benefits derived from the initial RCT intervention.

Factor	Level	Study cohort at long-term follow-up (*n* = 160)		
		Initial PR Non-completers	Initial PR Completers	*p*-value
*N*		30	130	
Characteristics at long-term follow-up				
Randomisation group				
	Physical Exercise Training (PExT)	17 (56.7%)	56 (43.1%)	0.18
	Singing for Lung Health (SLH)	13 (43.3%)	74 (56.9%)	
Age		69.6 (9.4)	73.2 (7.9)	0.03
BMI		28.2 (6.1)	29.4 (5.9)	0.33
Sex, Female		16 (53.3%)	46 (35.4%)	0.07
FEV1% predicted at initial RCT baseline		53.4 (20.1)	54.8 (15.4)	0.68
COPD-related medication, yes		26 (86.7%)	118 (90.8%)	0.48
Number of exacerbations within last year				
	0	15 (50.0%)	71 (62.8%)	0.35
	1–2	4 (13.3%)	36 (17.7%)	
	3 or more	11 (36.7%)	23 (19.5%)	
COPD-related GP visits within last year				
	0	15 (50.0%)	72 (54.2%)	0.092
	1–2	5 (16.7%)	37 (28.5%)	
	3 or more	10 (33.3%)	21 (16.2%)	
COPD-related hospitalisations within last year				
	0	25 (83.3%)	99 (76.2%)	0.56
	1–2	3 (10.0%)	25 (19.2%)	
	3 or more	2 (6.7%)	6 (4.6%)	
Smoking since RCT participation				
	Never smoker	2 (6.7%)	7 (5.5%)	0.02
	Previous smoker	16 (53.3%)	100 (76.9%)	
	Current smoker	12 (40.0%)	23 (17.7%)	
If current smoker; smoking amount				
	<10 cigarettes per day	5 (45%)	14 (56%)	0.60
	10 or more per day	7 (55%)	9 (44%)	
Scoring at long-term follow-up				
SGRQ Total score		43.3 (15.2)	39.8 (16.7)	0.32
HADS Anxiety score		6.4 (4.0)	5.6 (2.3)	0.17
HADS Depression score		5.3 (3.0)	4.3 (1.8)	0.02
mMRC Dyspnoea score		2.8 (1.1)	2.4 (1.1)	0.10
Long-term attendance in exercise training or lung choir (missing answers from *n* = 3)				
Have you been engaged in exercise training or lung choir within the last six months?				
	No attendance	22 (75.9%)	69 (53.9%)	0.03
	Attendance	7 (24.1%)	59 (46.1%)	
Overall evaluation of the initial RCT intervention (PExT/SLH)				
Satisfaction with the intervention				
	Not at all	5 (13.8%)	2 (1.5%)	<0.001
	To a small to moderate degree	9 (31.0%)	20 (15.4%)	
	To a high degree	16 (55.2%)	108 (83.1%)	
Experience that the intervention met disease-specific needs				
	Not at all	5 (13.8%)	2 (1.5%)	<0.01
	To a small to moderate degree	9 (31.0%)	28 (21.5%)	
	To a high degree	16 (55.2%)	100 (76.9%)	
Experience of relevance of the intervention				
	Not at all	5 (13.8%)	1 (0.8%)	<0.001
	To a small to moderate degree	9 (31.0%)	24 (18.5%)	
	To a high degree	16 (55.2%)	105 (80.8%)	
Experienced integration of tools and benefits from initial RCT				
Improved breathing control		2 (6.7%)	49 (37.7%)	<0.01
Improved management of dyspnoea		3 (10.0%)	34 (26.2%)	0.06
Improved physical strength		0 (0.0%)	7 (5.4%)	0.19
Improved physical fitness		1 (3.3%)	5 (3.8%)	0.89
Improved speaking/singing voice		0 (0.0%)	2 (1.5%)	0.49
Experienced no improvements		13 (43.3%)	50 (38.5%)	0.62

Data are presented as mean ± SD or number (%). BMI, Body Mass Index; FEV1% predicted, Forced expiratory volume in 1 s (FEV1), % of predicted at initial RCT baseline; SGRQ Total Score, St George's Respiratory Questionnaire; HADS, Hospital Anxiety and Depression Scale; Sub-scores, symptoms of anxiety (HADS-A) and depression (HADS-D); mMRC, modified Medical Research Council dyspnoea score. Differences between-groups were tested using Student's *t*-test (two-tailed), paired-samples *t*-test, χ^2^, or Fischer's exact test. Statistical analyses were performed using statistical software STATA 18 (StataCorp LLC, Texas, USA). Statistical significance was reached at *p* < 0.05.

##### Primary variable of interest

1. *Self-reported current attendance*

Current attendance was defined as having attended supervised exercise training (=with a trainer present) or a lung choir (18, 19) twice a month or more vs. less than twice a month within all of the past six months (self-reported). We dichotomised attendance as “Attendance” (i.e., twice a month or more) or “No attendance” (i.e., less than twice a month).

##### Secondary variables of interest

2. *Patient-reported outcomes*
  (a) Current status of QoL [measure: St George's Respiratory Questionnaire (SGRQ Total score) (20, 21)], widely applied in PR and maintenance research (8, 22).

To supplement, we included:

(b) Current symptoms of anxiety and depression (measure: Hospital Anxiety and Depression Scale (HADS); subscores: anxiety (HADS-A); depression (HADS-D) (23)). Current symptoms of anxiety and depression (measure: Hospital Anxiety and Depression Scale (HADS); subscores: anxiety (HADS-A); depression (HADS-D) (23)).
(c) Current level of dyspnoea (measure: Modified Medical Research Council Dyspnoea Scale (mMRC)) (24).

3. *Researcher-developed questionnaire about the perceived value and benefits of participating in the initial RCT*.
  (a) Overall evaluation of the initial RCT intervention (retrospectively): Participants’ overall satisfaction, alignments with needs, and experience of relevance; trichotomised in “Not at all”, “To a small to moderate degree”, and “To a high degree”.
  (b) Experience of tools and benefits (derived from the initial RCT) at present: Control over breathing, management of dyspnoea, physical strength, physical fitness, and/or singing/speaking voice; all dichotomised in “Yes” or “No”.

The researcher-developed questionnaire (please see Supplementary Figure S1) had been developed by primary study investigator (MK) and DB, based on variables of interest from the RCT (12, 16, 17) and aspects related to engagement in exercise training or lung choir, with specific focus on subjective outcomes (2, 22, 25, 26). Before onset of data collection, questions were discussed and settled with all research group members, and the questions and the overall data collection procedure were tested in a face-validity procedure in two participants and were well-accepted.

##### Variables related to primary study hypothesis

A. ***PR completion status during the initial RCT***

PR completion status during the initial RCT was defined as “PR Non-completer” if the study participant had dropped out during the RCT, and “PR Completer” if the participant had attended and had completed both RCT assessment procedures (at baseline and at follow-up).

##### Variables related to secondary study hypotheses (besides scoring at present)

B. ***Initial RCT randomisation***

Initial RCT randomisation reflected either having been randomised to PExT or SLH as part of the 10 weeks’ PR programme in the initial RCT (12). For further information about the initial RCT, including inclusion criteria, randomisation procedure, and content of the two study arms, please see the main RCT article and its supplementary materials (12).

C. ***PR adherence level during the initial RCT***

Level of PR adherence in the initial RCT was calculated ranging from 0 to 20 sessions attended and dichotomised as either “Low-moderate adherence” (0%–74% attendance) or “High adherence” (≥75% attendance).

### Analysis

Plan for analysis was discussed among MK, DB, and a biostatistician from Open Patient Data Explorative Network (OPEN), Odense University Hospital and Department of Clinical Research, University of Southern Denmark, before upload of statistical analysis plan.

#### Descriptive analyses

We performed stratified analyses to investigate characteristics and study outcomes (current attendance, scoring, and perceived value and benefits of participating in the initial RCT) at long-term follow-up in (1) “PR Non-completers” vs. “PR Completers” (Table 1); (2) “No attendance” vs. “Attendance” (Table 2); and (3) Initial RCT randomisation: “PExT” vs. “SLH” (Table 3).

**Table 2:** Attendance in exercise training or lung choir at present related to characteristics, quality of life, symptoms of anxiety and depression, dyspnoea, and perceived benefits derived from the initial RCT intervention.

Factor	Level	Study cohort at long-term follow-up (*n* = 157)		
		No attendance	Attendance	*p*-value
*N*		91	66	
Characteristics at long-term follow-up				
Randomisation group				
	Physical Exercise Training (PExT)	46 (50.6%)	27 (40.9%)	0.23
	Singing for Lung Health (SLH)	45 (49.4%)	39 (59.1%)	
Age		72.5 (7.4)	73.2 (6.6)	0.23
BMI at initial RCT baseline		29.1 (6.2)	29.5 (5.6)	0.69
Sex, Female		37 (40.6%)	23 (34.9%)	0.46
FEV1% predicted at initial RCT baseline		55.2 (15.5)	53.1 (17.3)	0.44
COPD-related medication, yes		81 (89.0%)	60 (90.9%)	0.70
Number of exacerbations within last year				
	0	51 (56.0%)	38 (57.6%)	0.39
	1–2	15 (16.5%)	17 (25.8%)	
	3 or more	25 (27.5%)	11 (16.7%)	
COPD-related GP visits within last year				
	0	52 (57.1%)	35 (53.0%)	0.08
	1–2	19 (20.9%)	23 (34.9%)	
	3 or more	20 (22.0%)	8 (12.1%)	
COPD-related hospitalisations within last year				
	0	75 (82.4%)	49 (74.2%)	0.18
	1–2	11 (12.1%)	15 (22.7%)	
	3 or more	5 (5.5%)	2 (3.0%)	
Smoking since RCT participation				
	Never smoker	4 (4.4%)	6 (9.1%)	0.22
	Previous smoker	65 (71.4%)	51 (77.3%)	
	Current smoker	22 (24.2%)	9 (13.6%)	
If current smoker; smoking amount				
	<10 cigarettes per day	8 (36.4%)	7 (77.9%)	0.06
	10 or more per day	14 (64.6%)	2 (22.1%)	
Scoring at long-term follow-up				
SGRQ Total score		43.1 (16.7)	36.9 (15.4)	0.02
HADS Anxiety score		6.1 (3.0)	5.2 (2.0)	0.04
HADS Depression score		4.8 (2.3)	4.0 (1.7)	0.02
mMRC Dyspnoea score		2.5 (1.1)	2.4 (1.0)	0.35
Overall evaluation of the initial RCT intervention (PExT/SLH)				
Satisfaction with the intervention				
	Not at all	5 (5.5%)	1 (1.6%)	0.36
	To a small to moderate degree	18 (19.8%)	11 (16.7%)	
	To a high degree	68 (74.7%)	54 (81.8%)	
Experience that the intervention met disease-specific needs				
	Not at all	5 (5.5%)	1 (1.6%)	0.33
	To a small to moderate degree	18 (19.8%)	17 (25.8%)	
	To a high degree	68 (74.7%)	48 (72.7%)	
Experience of relevance of the intervention				
	Not at all	5 (5.5%)	0 (0.0%)	0.15
	To a small to moderate degree	17 (18.7%)	14 (21.2%)	
	To a high degree	69 (75.8%)	52 (78.8%)	
Experienced integration of tools and benefits from initial RCT				
Improved breathing control		26 (28.6%)	25 (37.9%)	0.22
Improved management of dyspnoea		18 (19.8%)	19 (28.8%)	0.19
Improved physical strength		5 (5.5%)	2 (3.0%)	0.46
Improved physical fitness		1 (1.1%)	5 (7.6%)	0.04
Improved speaking/singing voice		2 (2.2%)	0 (0.0%)	0.23
Experienced no improvements		40 (44.0%)	23 (34.9%)	0.25

Data are presented as mean ± SD or number (%). BMI, Body Mass Index; FEV1% predicted, Forced expiratory volume in 1 s (FEV1), % of predicted at initial RCT baseline; SGRQ Total Score, St George's Respiratory Questionnaire; HADS, Hospital Anxiety and Depression Scale; Sub-scores, symptoms of anxiety (HADS-A) and depression (HADS-D); mMRC, modified Medical Research Council dyspnoea score. Differences between-groups were tested using Student's *t*-test (two-tailed), paired-samples *t*-test, χ^2^, or Fischer's exact test. Statistical analyses were performed using statistical software STATA 18 (StataCorp LLC, Texas, USA). Statistical significance was reached at *p* < 0.05.

**Table 3:** Initial RCT randomisation arm related to characteristics, attendance in exercise training or lung choir, quality of life, symptoms of anxiety and depression, dyspnoea, and perceived benefits derived from the initial RCT intervention.

Factor	Level	Study cohort at long-term follow-up (*n* = 160)		
		Initial RCT randomisation: PExT	Initial RCT randomisation: SLH	*p*-value
*N*		73	87	
Characteristics at long-term follow-up				
Age		71.8 (7.2)	73.2 (8.3)	0.42
BMI at initial RCT baseline		29.0 (5.5)	29.4 (6.3)	0.73
Sex, Female		23 (32.0%)	39 (45.0%)	0.09
FEV1% predicted at initial RCT baseline		57.0 (17.1)	52.4 (15.4)	0.08
COPD-related medication, yes		66 (90.0%)	75 (89.0%)	0.82
Number of exacerbations within last year				
	0	40 (54.8%)	46 (52.9%)	0.83
	1–2	18 (24.7%)	12 (14.0%)	
	3 or more	15 (20.6%)	18 (20.7%)	
COPD-related GP visits within last year				
	0	41 (56.2%)	46 (52.9%)	0.98
	1–2	19 (26.0%)	26 (29.9%)	
	3 or more	13 (17.8%)	15 (17.2%)	
COPD-related hospitalisations within last year				
	0	58 (79.5%)	66 (75.9%)	0.78
	1–2	11 (15.1%)	17 (20.7%)	
	3 or more	4 (5.5%)	5 (5.8%)	
Smoking since RCT participation				
	Never smoker	5 (6.9%)	7 (8.1%)	0.84
	Previous smoker	54 (74.0%)	65 (74.7%)	
	Current smoker	14 (19.2%)	15 (17.2%)	
If current smoker; smoking amount				
	<10 cigarettes per day	9 (64.0%)	6 (40%)	0.19
	10 or more per day	5 (36.0%)	9 (60%)	
Scoring at long-term follow-up				
SGRQ Total score		39.2 (15.6)	41.5 (17.1)	0.41
HADS Anxiety score		5.7 (2.8)	5.8 (2.6)	0.80
HADS Depression score		4.5 (2.1)	4.4 (2.2)	0.66
mMRC Dyspnoea score		2.3 (1.0)	2.6 (1.1)	0.10
Long-term attendance in exercise training or lung choir (missing answers from *n* = 3)				
Have you been engaged in exercise training or lung choir within the last six months?				
	No attendance	46 (63.0%)	45 (54.0%)	0.23
	Attendance	27 (37.0%)	39 (46.0%)	
Overall evaluation of the initial RCT intervention (PExT/SLH)				
Satisfaction with the intervention				
	Not at all	4 (5.5%)	2 (2.3%)	0.60
	To a small to moderate degree	13 (17.8%)	17 (19.5%)	
	To a high degree	56 (76.7%)	68 (64.3%)	
Experience that the intervention met disease-specific needs				
	Not at all	4 (5.5%)	2 (2.3%)	0.60
	To a small to moderate degree	16 (21.9%)	20 (22.9%)	
	To a high degree	53 (72.6%)	65 (74.7%)	
Experience of relevance of the intervention				
	Not at all	4 (5.5%)	1 (1.2%)	0.23
	To a small to moderate degree	16 (21.9%)	16 (18.4%)	
	To a high degree	53 (72.6%)	70 (80.5%)	
Experienced integration of tools and benefits from initial RCT				
Improved breathing control		16 (21.9%)	35 (40.2%)	0.01
Improved management of dyspnoea		12 (16.1%)	25 (29.1%)	0.07
Improved physical strength		6 (8.2%)	1 (1.2%)	0.03
Improved physical fitness		5 (7.4%)	1 (1.2%)	0.06
Improved speaking/singing voice		0 (0.0%)	2 (2.3%)	0.19
Experienced no improvements		12 (16.2%)	25 (29.1%)	0.46

Data are presented as mean ± SD or number (%). BMI, Body Mass Index; FEV1% predicted, Forced expiratory volume in 1 s (FEV1), % of predicted at initial RCT baseline; SGRQ Total Score, St George's Respiratory Questionnaire; HADS, Hospital Anxiety and Depression Scale; Sub-scores, symptoms of anxiety (HADS-A) and depression (HADS-D); mMRC, modified Medical Research Council dyspnoea score. Differences between-groups were tested using Student's *t*-test (two-tailed), paired-samples *t*-test, χ^2^, or Fischer's exact test. Statistical analyses were performed using statistical software STATA 18 (StataCorp LLC, Texas, USA). Statistical significance was reached at *p* < 0.05.

#### Sub-group analyses

To check comparability, we performed stratified sub-analyses of initial RCT characteristics and performance in initial PR Non-completers vs. initial PR Completers (Supplementary Table S1) and of Low-moderate initial PR adherence vs. High initial PR adherence (Supplementary Table S2). In addition, we explored: Initial RCT characteristics in Living participants vs. Deceased participants in the initial RCT (Supplementary Table S3).

Continuous data were described as either mean ± standard deviation and categorial data were described with number and percentage. Differences between-groups were tested using Student's *t*-test (two-tailed), paired-samples *t*-test, *χ*^2^, or Fischer's exact test. Statistical analyses were performed using statistical software STATA 18 (StataCorp LLC, Texas, USA). We did not employ imputation but reported any missing data transparently. An alpha level of *p* ≤ 0.05 was adopted for statistical significance.

STROBE Statement checklist for cohort studies was consulted for reporting.

## Results

### Participants

Time since the post-assessment in the initial RCT was 56 months (range: 45–62 months; mean and median were identical) with the majority of the 29 initial RCT clusters (*n* = 23; 79.3%) within the time span of 56 months ± 6 months) (see Supplementary Figure S2 for an overview of time since post-assessment for all of the 29 clusters in the initial RCT) (12).

**Figure 1:**
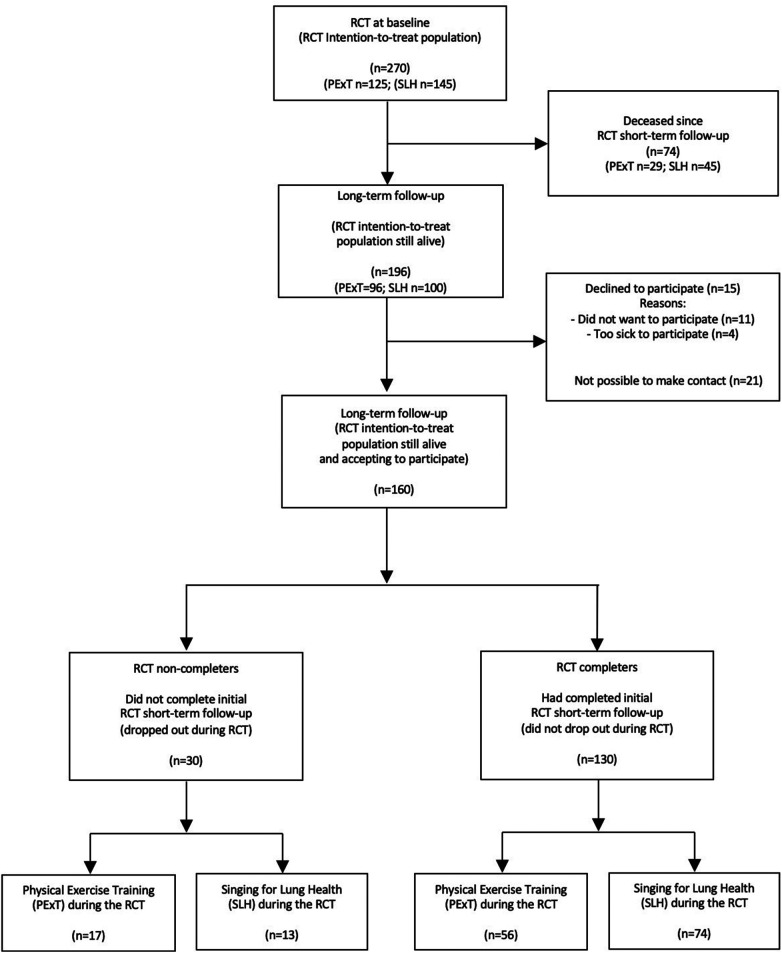
Consort flow diagram.

Figure 1 shows the flow of study participants included in the present study. In total, 74 (27%) out of the initial 270 RCT study participants were deceased. Out of the 196 living participants, 15 (8%) declined to participate, 21 (11%) did not respond across three contact attempts, and, thus, 160 (82%) of living RCT participants were included. In total, 30 participants (19% of 160) had not completed the post-assessment after the initial PR programme (=“PR Non-completers”). Compared to living participants, those who were deceased since initial RCT baseline had been more challenged, e.g., with higher age, lower lung function and walking distance, and fewer were co-habiting (see Supplementary Table S3).

### Results related to primary study hypothesis

(1) *Initial PR completion status related to current self-reported attendance, scoring, and perceived benefits*

Table 1 shows that initial PR Non-completers (*n* = 30) had a lower level of self-reported current attendance in exercise training or a lung choir as compared to initial PR Completers (*n* = 130) (24% vs. 46%; *p* = 0.03), whereas QoL score was not statistical significantly different between the groups (*p* = 0.32).

Initial Non-completers were characterised by being more frequently smokers at present (12 (40%) vs. 23 (18%); *p* = 0.02). They also displayed more symptoms of depression at present (mean 5.3 ± 3.0 vs. 4.3 ± 1.8; *p* = 0.02), had overall been less satisfied (retrospectively) with the initial intervention (for all three domains: *p *≤ 0.001), and experienced significantly less control over their breathing, compared to initial PR Completers (7% vs. 38%; *p* ≤ 0.01). There was no difference in distribution in age, BMI, or lung function (FEV1% predicted) at initial RCT baseline, or in having participated in PExT or SLH as intervention as part of PR during the initial RCT (*p* = 0.18).

### Results related to secondary study hypotheses

(2) *Self-reported current attendance related to scoring and to perceived benefits*

Regarding current attendance and QoL score, Table 2 shows that those who reported to current attendance also reported to have a better QoL score (SGRQ; “Attendance”: mean 36.9 ± 15.4 vs. “No attendance” 43.1 ± 16.7; *p* = 0.02) and, moreover, had lower anxiety and depression scores at present (HADS-A: *p* = 0.04, HADS-D: *p* = 0.02) compared to those with “No attendance”. No specific characteristics or perceived benefits were related to “No attendance” or “Attendance”. For this analysis, three observations in the study cohort had missing data (i.e., number included in the analysis: *n* = 157) (Table 2).

(3) *Initial randomisation related to current self-reported attendance, scoring, and perceived benefits*

Table 3 shows that across initial randomisation arms, PExT (*n* = 73; 46%) vs. SLH (*n* = 87; 54%), there were no significant between-group differences in current self-reported attendance (initial randomisation to PExT, “Attendance”: 27 (37%); initial randomisation to SLH, “No attendance”: 39 (46%); *p* = 0.23). Moreover, there were no differences in current QoL score, HADS, and mMRC scores, nor in the overall evaluation of the initial intervention (retrospectively). However, a larger proportion of participants having had SLH as part of PR in the initial RCT reported to have improved breathing control (PExT: 22%, SLH: 40%; *p* = 0.01).

(4) *PR adherence level in the initial RCT related to current self-reported attendance, scoring, and perceived benefits*

Adherence level during the initial RCT (Supplementary Table S2) was not related to any characteristics or outcomes in the present study.

#### Subgroup analyses

At the time of the initial RCT (Supplementary Table S1), PR Non-completers were more often smokers than PR Completers (43% vs. 20%; *p* = 0.02) and had poorer SGRQ score at initial RCT baseline than those who had completed the RCT (SGRQ Total score; mean 50.1 ± 18.3 vs. 42.7 ± 17.2; *p* = 0.04).

## Discussion

This observational long-term follow-up study suggests that current self-reported attendance in exercise training or a lung choir and QoL status at present are related to having completed an initial pulmonary rehabilitation (PR) programme around four years previously, regardless of initial randomisation to conventional physical exercise training (PExT) or singing as training modality (Singing for Lung Health (SLH)) or to initial PR adherence level.

### Results related to primary study hypothesis

(1) *Initial PR completion status related to current self-reported attendance, scoring, and perceived benefits*

Little is overall known about the long-term impact of initial PR completion status (2). However, one of the most significant findings in our present study was that those who did not complete the initial PR programme were less likely to engage in long-term exercise training or participate in a lung choir at present compared to those who completed initial PR (Table 1). In addition, initial PR Non-completers reported (retrospectively) to having been less satisfied with the initial intervention, seemed to experience less control over their breathing compared to initial PR Completers, and were more challenged overall than initial Completers (Table 1). This is in keeping with the findings in our previous RCT (12) that those who did not complete the PR programme were characterised by a more challenged starting point (12, 16). Furthermore, this underlines a need for special attention in following up participants who have a more challenged starting point at PR onset, and in following up those who do not find the PR programme motivating or relevant, and those who drop out during the PR programme, in order to support these people in improving their levels of activity and QoL.

### Results related to secondary study hypotheses

(2) *Current self-reported attendance related to scoring and perceived benefits*

Study participants in the initial PR programme (12) had been encouraged to maintain an active lifestyle autonomously to avoid the previously reported fade away of effects of PR (3, 7–9). However, there was no standard procedure described for this and no standard, structured offers available locally after PR. In the present study, we considered both self-reported attendance in supervised exercise training or lung choir as “attendance” (i.e., with a professional trainer or singing teacher/choir director present), potentially bridging to each of the interventions which participants were initially introduced to (12).

Given the lack of an available structured maintenance model after initial PR (2, 3, 7, 8), given the heterogeneity of current lung choirs (18), and given that our previous RCT design did not include a structured follow-up, the quality and content of any available local offers were, unclear. Moreover, we chose to report “attendance” as a dichotomous variable, interpreting “attendance” if study participants had attended twice a month or more during all of the past six months. However, the study consistently suggests that attendance was related to both scoring at present in QoL and in symptoms of anxiety and depression. Although our study is not able to elucidate any underlying causality, this finding seems to support previous evidence that supervised exercise training following PR may preserve QoL (5, 6, 25). It would be interesting to investigate long-term attendance and preservation of effects in settings with more structured and well-defined offers and with reporting in a more detailed manner to clarify the ideal maintenance model and aspects related to motivation and adherence.

Overall, previous studies on the impact of supervised exercise training on long-term maintenance of effects have been heterogeneous regarding population, content, setting, frequency, exercise intensity, study duration, and bias (7, 8). Findings from studies vary (3, 7, 8, 27), maintenance training once a week may preserve effects up to two years after initial PR (28, 29). A Cochrane review with 21 studies (Malaguti et al.) (8) reports only low-moderate evidence for supervised physical training in preserving exercise capacity and QoL after initial PR, but a recent Clinical Practice Guideline (Rochester) (3) suggest either supervised maintenance PR or usual care after initial PR for COPD (3). The findings from the present study concur with the previously reported correlation between attendance in supervised physical exercise training and QoL (3, 7, 8) as well as with the recent recommendations regarding sustained support after PR (2, 4).

(3) *Initial randomisation related to current self-reported attendance, scoring at present, and to perceived benefits*

The present study found no differences between initial randomisation arms (PExT or SLH) in current long-term self-reported attendance or in scoring at present (Table 3). This seems to be in line with the findings in the initial RCT (12) in which SLH conferred measurable short-term improvements in walking distance and QoL comparable to PExT, in a dose-response manner in both initial study arms (12, 16). Regarding the perceived benefits of the intervention at present (retrospectively), initial randomisation to SLH interestingly seemed to be related to long-term improved breathing control at present (Table 3). This finding corresponds well with the *post hoc* analyses from the RCT, suggesting that SLH was associated with improved dyspnoea control and inspiratory muscle strength and control (17). It would be relevant to explore effects and mechanisms related to these aspects in future short-term and long-term studies.

Our study is the first to explore the indicated impact of a PR programme including SLH as training modality on long-term attendance and QoL score at present, although it was outside the scope of our previous RCT to report on long-term follow-up status. Initially, in planning of the present study, we considered relating the long-term follow-up data directly to the RCT data. However, we concluded that this would have required a structured follow-up process to justify such analyses of correlations, as the many unfamiliar confounding factors would easily lead to an over-exaggeration of any observed effect, impact, and causality. Therefore, we chose to only report on self-reported attendance within the past six months and on QoL score at present.

Notably, in our present study, initial randomisation to SLH did not seem to limit long-term attendance in either exercise training or lung choir activity or to affect QoL score at present. This is in support of SLH as a safe and relevant activity (30) and is in keeping with a previous service evaluation, suggesting positive long-term implications of a SLH course (15). Further studies are needed, however, to clarify any long-term implications, both of singing as training modality as part of PR and within any maintenance model.

(4) *PR adherence level in the initial RCT related to current self-reported attendance, scoring, and perceived benefits*

In the planning of our present study, we anticipated that those having experienced an effect during the initial PR programme would be more likely to demonstrate current attendance in exercise training or lung choir activity, based on a rationale related to the significant dose-response relationship demonstrated in our previous RCT (12, 16). However, surprisingly, no factors at present were related to the initial PR adherence level (see Supplementary Table S2), suggesting that initial PR adherence level neither predicts nor limits current attendance or QoL score at present (i.e., long-term after an initial PR programme). It would be interesting to explore the long-term implications of PR adherence level in future studies.

### Implications for practice and research – with specific emphasis on singing as activity

Both the present study and our initial RCT address the stated request for further investigation of motivating and relevant non-pharmacological interventions as part of an increasingly personalised PR-offer (1, 2, 4, 31). The present study provides unique insights into the long-term lifestyle behaviour of participants after an initial PR programme in a real-life setting without a formal offer to support long-term maintenance of an active life-style. Our present study underlines the need for establishing local supervised support for people with COPD after PR, as long-term attendance seems to be related to preserving effects of PR programmes. Future short-term studies should ideally include both a maintenance strategy with diverse activities and with structured, long-term follow-up assessments. Such research could provide important knowledge to build a relevant and sustainable maintenance model after PR, not at least to support both those with resources and those with a more challenged starting point.

We believe that there are several aspects in favour of pursuing further research in singing as a potentially relevant activity, both as part of PR and within a sustainable maintenance model. Overall, group singing for people with respiratory diseases has become increasingly popular in many countries worldwide (10, 18, 32–34), and, thus, seems to be a motivating community-based activity. In addition, an increasing number of studies have initially investigated physiological and psychosocial short-term outcomes of different types of singing interventions, although these studies are characterised by heterogeneity and inconsistent findings (10, 13, 18, 30, 35–39). Qualitative studies have, moreover, reported benefits of community-based singing groups regarding physiological and psychological aspects for the individual, but also regarding singing as a means to facilitate social cohesion and long-term participation (14, 18, 35). A Cochrane review (McNamara et al.) (30), based on three initial, small RCTs, found, however, low to very low quality of evidence that singing may improve physical health, but found no impact on dyspnoea or respiratory-specific quality of life. An update of this review would be of interest, with inclusion of more recently conducted RCTs and with inclusion of all types of respiratory diseases (17, 33, 40–42).

However, group singing for people with respiratory diseases continues to be delivered heterogeneously and without any standard disease-specific framework or investigation of effects (18, 19), whereas SLH is a well-defined approach and could be considered current best-practice. SLH has been systematically developed as a holistic approach within a multidisciplinary health-care setting since 2007 (10) and includes customised and structured exercises and songs as “tools for purpose” to support breathing and vocal expression and includes additional movement and dancing (10, 11, 13, 14). However, SLH requires a thorough disease-specific understanding, training, and supervision and is so far only accessible in English, which limits availability, diffusion, and implementation. Training facilitated by the SLH team has previously been offered for singing leaders by the British Lung Foundation and was found to be beneficial (14, 18). A cross-national, cross-cultural, and cross-disciplinary strategy for providing and further developing SLH (or similar framework) as a standardised training programme should be encouraged to ensure both ongoing knowledge sharing, network, and supervision. In addition, this would (1) increase the possibility of conducting further rigorously-driven studies to clarify the potential of singing as a relevant and validated activity as part of PR and/or as part of a future maintenance model through qualified and ongoing documentation, research, and evaluation; (2) Ensure predictable outcomes for the participants and for health-professionals considering recommending singing as an activity for their patients; (3) Ensure optimal content and delivery and benefit the facilitators who may struggle with feelings of isolation, insecurity, and inadequacy without having a solid framework (18) and who often even struggle with work-related and severe mental health issues (43).

Obviously, exercise training continues to be the best documented modality to preserve long-term exercise capacity and QoL (3, 7, 8), but it would be interesting to investigate whether a structured programme of singing might serve as a motivating offer to support an active lifestyle for those who do not prefer exercise training, either in its own right or combined with supervised exercise training. Further studies are, evidently, needed, e.g., to explore whether an active choice of PR with SLH would impact completion and adherence rates, QoL, and other outcomes of interest positively during PR. Moreover, future studies should include structured long-term follow-up assessments and documentation, employing both key objective and subjective outcomes in PR, should be conducted both in settings with and without structured maintenance models, and should e.g., explore singing as part of the model. In the near future, results from an ongoing randomised controlled feasibility study by Lewis et al., aiming to explore the impact of SLH after PR (although only within a 12 weeks’ follow-up course) (44), are anticipated with interest.

### Strengths and limitations of the study

The present study has a number of strengths: It is the first study to investigate long-term current engagement in activity and QoL score at present related to completion status in an initial PR programme, and, thus, the study may provide important clinical perspectives regarding the need for special attention targeted this group of PR participants. Secondly, it is the first study to propose implications on current attendance and QoL score long-term after a PR programme with SLH and may inspire the conduct of future structured studies on long-term implications of SLH. Thirdly, the present study is based on a real-life setting regarding current, *de facto* available offers of exercise training or lung choir after PR, which may strengthen the generalisability of our findings. Fourthly, the study was based on a cohort without specific preference for singing beforehand, as RCT participants were originally referred for PR with PExT, which may strengthen the validity and trustworthiness of our findings. Fifthly, we managed to recruit a large proportion among living study participants from our initial RCT cohort, and the present study cohort was comparable to the RCT-cohort, which may have reduced the risk of sampling and selection bias.

The study, however, also has a number of limitations. It was an observational study with no structured intervention or additional data collection since the initial RCT, which may have led to design bias. Secondly, the study did not include data from the full initial study sample, which may have led to selection bias. Thirdly, the study was based only on self-reported and subjective data which may have led to design and recall bias. Fourthly, previous maintenance studies have been one to three years since original PR, whereas the timeframe since RCT termination in the present study was more than four years (mean/median: 56 months) and with a varying range between 45 and 62 months, which may have led to design and recall bias, and which may compromise the validity and generalisability of our findings. Fifthly, we lack information about type, quality, intensity, dose, and setting of both the self-reported supervised exercise training and lung choir participation, which may have led to design and recall bias.

Taken together, however, we believe that our present study provides interesting perspectives and guidance for future practice and research. Further work is needed to guide the development of increasingly personalised PR programmes and sustainable maintenance models to support an active lifestyle and the preservation of QoL after PR, for people living with respiratory diseases, including COPD.

## Conclusion

Our study suggests that long-term, current attendance in exercise training or lung choir activity and status of quality of life is positively related to initial completion of a pulmonary rehabilitation (PR) programme for people with COPD, based on current self-reported data from around four years after initial PR. Attending a course with singing (Singing for Lung Health with no physical exercise training as part of PR) did not seem to limit long-term current attendance in exercise training or lung choir or in quality of life scoring at present, nor did the initial PR adherence level. We suggest that future PR programmes give special attention to those who do not complete PR to support them in maintaining an active lifestyle and quality of life.

## Data Availability

De-identified data collected for the study will be available from September 2024, upon reasonable request.
