# Supplementary material for: Long-term self-reported attendance in exercise training or lung choir and status of quality of life following initial pulmonary rehabilitation for COPD
Source: Front Rehabil Sci. 2024 Sep 19;5:1447765. doi: 10.3389/fresc.2024.1447765 (PMC11446877; doi:10.3389/fresc.2024.1447765)
Supplement: Supplementary file 1 [file Datasheet1.pdf]

Supplementary materials for manuscript:

Long-term self-reported attendance in exercise training or lung choir and status of quality of life following pulmonary rehabilitation for COPD

Authors: Mette Kaasgaard, Uffe Bodtger, Søren T. Skou, Stephen Clift, Ole Hilberg, Daniel Bech Rasmussen, Anders Løkke.

Supplementary materials - content:

Page 2: Supplementary Figure 1: Specially developed questions about the perceived value and benefits of participating in the initial RCT.

Page 3: Supplementary Figure 2: Overview of time since post-assessment in the initial RCT.

Page 4: Supplementary Table 1: Characteristics and performance in initial RCT (non-completers vs completers).

Page 5: Supplementary Table 2: Characteristics, attendance in exercise training or lung choir, quality of life, symptoms of anxiety and depression, dyspnoea, and perceived benefits derived from the initial RCT intervention related to adherence level in the initial RCT.

Page 6: Supplementary Table 3: Characteristics and performance of living vs. deceased participants in the initial RCT.

Supplementary Figure 1: Specially developed questions about the perceived value and benefits of participating in the initial RCT

**Overall evaluation of the initial RCT intervention (PExT/SLH)**	
Question	*Response categories*
Looking back, how satisfied have you overall been with participating in the initial programme with either singing or physical exercise training?	• Not at all<br>• To a small to moderate degree<br>• To a high degree
Did you feel that the intervention (singing or physical exercise training) met your needs?	• Not at all<br>• To a small to moderate degree<br>• To a high degree
Looking back, how relevant do you think the initial programme with either singing or physical exercise training was to you?	• Not at all<br>• To a small to moderate degree<br>• To a high degree
**Experienced integration of tools and benefits from the initial RCT**	
Question	*Response categories*
Compared to before the initial programme with either singing or physical exercise training, have you experienced…	
Improvements in your breathing control?	Yes/No
Improvements in the way you are able to manage your dyspnoea?	Yes/No
Improvements in your overall physical strength?	Yes/No
Improvements in your overall physical fitness?	Yes/No
Improvements in your overall speaking/singing voice?	Yes/No
Experienced no improvements?	Yes/No

Supplementary Figure 2: Overview of time since post-assessment in the initial RCT

Month of post-assessment in initial RCT for all 29 clusters		Months to Feb 2023
Dec/17		62
Dec/17		62
Dec/17		62
Dec/17		62
Dec/17		62
Dec/17		62
Jan/18		61
Jan/18		61
Jan/18		61
Feb/18		60
Feb/18		60
May/18		57
May/18		57
Jun/18		56
Jun/18		56
Jun/18		56
Jul/18		55
Jul/18		55
Jul/18		55
Aug/18		54
Aug/18		54
Dec/18		50
Dec/18		50
Jan/19		49
Jan/19		49
Mar/19		47
Apr/19		46
May/19		45
May/19		45
	Sum months from RCT to Feb 2023	1611
	**Mean months** from RCT to Feb 2023	**56**
	**Median months** from RCT to Feb 2023	**56**

Supplementary Table 1: Characteristics and performance in initial RCT (non-completers vs completers)

Factor	Level	N=160		
		RCT Non-completers	RCT Completers	p-value
N		30	130	
**Characteristics and performance in initial RCT (at baseline)**				
**Randomisation group**				
	Physical Exercise Training (PExT)	17 (56.7%)	56 (43.1%)	0.18
	Singing for Lung Health (SLH)	13 (43.3%)	74 (56.9%)	
**Age**		64.9 (9.4)	68.5 (7.9)	0.03
**BMI**		28.2 (6.1)	29.4 (5.9)	0.33
**Sex, Female**		16 (53.3%)	46 (35.4%)	0.07
**FEV1 % predicted**				
**GOLD class**				
	1	2 (6.7%)	5 (3.9%)	0.08
	2	12 (40.0%)	76 (58.5%)	
	3	11 (36.7%)	43 (33.1%)	
	4	5 (16.7%)	6 (4.6%)	
**Smoking Status, n (%)**		39.2 (14.9)	42.3 (24.7)	0.53
	Never smoker	2 (6.7%)	8 (6.2%)	0.02
	Previous smoker	15 (50.0%)	96 (73.8%)	
	Current smoker	13 (43.3%)	26 (20.0%)	
**If previous or current smoker; pack years**		39.2 (14.9)	41.7 (23.9)	0.61
**Marital status, number, n (%)**				
	Married/co-habiting	21 (70.0%)	88 (67.7%)	0.81
**Income**				
	Low income	29 (96.7%)	123 (94.6%)	0.60
	Medium income	0 (0.0%)	4 (3.1%)	
	High income	1 (3.3%)	3 (2.3%)	
**Highest education**				
	Low education	20 (66.7%)	74 (56.9%)	0.45
	Medium education	10 (33.3%)	52 (40.0%)	
	High education	0 (0.0%)	4 (3.1%)	
**Living place**				
	Rural	20 (66.7%)	83 (63.8%)	0.70
	Medium	8 (26.7%)	42 (32.3%)	
	City	2 (6.7%)	5 (3.8%)	
**Occupational status**				
	Full- or part time job	4 (13.3%)	18 (13.8%)	0.89
	Unemployed/retired	26 (86.7%)	112 (86.2%)	
**Adherence to the intervention (calculated at RCT short-term follow-up)**				
	0-49%	27 (90.0%)	23 (17.7%)	<0.001
	50-74%	3 (10.0%)	107 (82.3%)	
**Performance and scoring at short-term follow-up in initial RCT (at baseline)**				
**6MWT distance**		388.7 (100.1)	410.8 (93.2)	0.26
**SGRQ Total score**		50.1 (18.3)	42.7 (17.2)	0.04
**HADS Anxiety score**		4.7 (3.5)	5.0 (3.9)	0.68
**HADS Depression score**		4.1 (3.4)	3.0 (3.1)	0.08
**mMRC Dyspnoea score**		2.3 (1.2)	1.8 (1.1)	0.052

Supplementary Table 1 text: Data are presented as mean ±SD or number (%). BMI: Body Mass Index. FEV1%predicted: forced expiratory volume in 1 second expressed as % of predicted; GOLD: Global Initiative for Chronic Obstructive Lung Disease. SGRQ Total Score: St George's Respiratory Questionnaire. HADS: Hospital Anxiety and Depression Scale; Sub-scores: symptoms of anxiety (HADS-A) and depression (HADS-D). mMRC: modified Medical Research Council dyspnoea score. Differences between-groups were tested using Student's t-test (two-tailed), paired-samples t-test, Chi^2, or Fischer's exact test. Statistical analyses were performed using statistical software STATA 18 (StataCorp LLC, Texas, USA). Statistical significance was reached at $p<0.05$.

Supplementary Table 2: Characteristics, attendance in exercise training or lung choir, quality of life, symptoms of anxiety and depression, dyspnoea, and perceived benefits derived from the initial RCT intervention related to adherence level in the initial RCT

		N=130		
**Factor**	**Level**	**Low-moderate RCT adherence (<74% attendance)**	**High RCT adherence (≥ 75% attendance)**	**p-value**
**N**		**23**	**107**	
**Characteristics at long-term follow-up**				
**Randomisation group**				
	Physical Exercise Training (PExT)	11 (47.8%)	45 (42.1%)	0.61
	Singing for Lung Health (SLH)	12 (52.2%)	62 (57.9%)	
**Age**		70.7 (6.9)	73.8 (7.2)	0.13
**BMI**		31.2 (5.3)	29.0 (6.0)	0.11
**Sex, Female**		8 (34.8%)	38 (35.5%)	0.95
**COPD-related medication**				
	No	3 (13.6%)	9 (8.5%)	0.45
	Yes	19 (86.4%)	97 (91.5%)	
**Number of exacerbations within last year**				
	0	12 (67%)	59 (62%)	0.72
	1 to 2	2 (11%)	18 (19%)	
	3 or more	4 (22%)	18 (19%)	
**COPD-related GP visits within last year**				
	0	13 (59.1%)	59 (55.7%)	0.75
	1 to 2	5 (22.7%)	32 (30.2%)	
	3 or more	4 (18.2%)	15 (14.2%)	
**COPD-related hospitalisations within last year**				
	0	17 (77.3%)	82 (77.4%)	1.00
	1 to 2	4 (18.2%)	19 (17.9%)	
	3 or more	1 (4.5%)	5 (4.7%)	
**Smoking since RCT participation**				
	Never smoker	2 (9.1%)	5 (4.8%)	0.70
	Previous smoker	17 (77.3%)	83 (79.0%)	
	Current smoker	3 (13.6%)	17 (16.2%)	
**If current smoker; smoking amount**				
	<10 cigarettes per day	2 (67%)	8 (53%)	0.67
	10 or more per day	1 (33%)	7 (47%)	
**Scoring at long-term follow-up**				
**SGRQ Total score**		36.2 (18.2)	40.4 (16.4)	0.33
**HADS Anxiety score**		5.3 (2.3)	5.7 (2.3)	0.43
**HADS Depression score**		3.9 (2.3)	4.4 (1.8)	0.26
**mMRC Dyspnoea score**		2.4 (1.3)	2.4 (1.0)	0.95
**Long-term attendance in exercise training or lung choir**				
**Have you been engaged in exercise training or lung choir within the last six months?**				
	No attendance	10 (45.5%)	59 (55.7%)	0.38
	Attendance	12 (54.5%)	47 (44.3%)	
**Overall evaluation of the initial RCT intervention (PExT/SLH)**				
**Satisfaction with the intervention**				
	Not at all	0 (0.0%)	2 (1.9%)	0.22
	To a small to moderate degree	6 (27.3%)	14 (13.2%)	
	To a high degree	16 (72.7%)	90 (84.9%)	
**Experience that the intervention met disease-specific needs**				
	Not at all	0 (0.0%)	2 (1.9%)	0.56
	To a small to moderate degree	6 (27.3%)	20 (18.9%)	
	To a high degree	16 (72.7%)	84 (79.2%)	
**Experience of relevance of the intervention**				
	Not at all	0 (0.0%)	1 (0.9%)	0.36
	To a small to moderate degree	6 (27.3%)	16 (15.1%)	
	To a high degree	16 (72.7%)	89 (84.0%)	
**Experienced integration of tools and benefits from initial RCT**				
Improved breathing control		8 (34.8%)	41 (38.3%)	0.75
Improved management of dyspnoea		5 (21.7%)	29 (27.1%)	0.60
Improved physical strength		0 (0.0%)	7 (6.5%)	0.21
Improved physical fitness		0 (0.0%)	5 (4.7%)	0.29
Improved speaking/singing voice		1 (4.3%)	1 (0.9%)	0.23
Experienced no improvements		10 (43.5%)	40 (37.4%)	0.59

Supplementary 2 Table text: Data are presented as mean ±SD or number (%). BMI: Body Mass Index. SGRQ Total Score: St George's Respiratory Questionnaire. HADS: Hospital Anxiety and Depression Scale; Sub-scores: symptoms of anxiety (HADS-A) and depression (HADS-D). mMRC: modified Medical Research Council dyspnoea score. Differences between-groups were tested using Student's t-test (two-tailed), paired-samples t-test, Chi^2, or Fischer's exact test. Statistical analyses were performed using statistical software STATA 18 (StataCorp LLC, Texas, USA). Statistical significance was reached at $p<0.05$.

Supplementary Table 3: Characteristics and performance of living vs. deceased participants in the initial RCT cohort

	Initial RCT cohort (n=270)		
	Living participants at Long-term follow-up	Participants deceased since RCT	p-value for difference between-groups
**N**	**196**	**74**	
**RCT randomisation, n (%)**			
Singing for Lung Health (SLH)	100 (69.0%)	45 (31.0%)	0.15
Physical Exercise Training (PExT)	96 (77.0%)	29 (23.0%)	
**SOCIODEMOGRAPHICS - RCT baseline**			
**Age**	68.2 (8.5)	73.1 (7.2)	<0.001
**Sex (female), n (%)**	120 (61.2%)	48 (64.9%)	0.58
**BMI**	28.7 (5.7)	25.8 (6.3)	<0.001
**Educational level, n (%)**			
Low education	115 (58.7%)	47 (63.5%)	0.69
Medium education	76 (38.8%)	26 (35.1%)	
High education	5 (2.6%)	1 (1.4%)	
**Occupational status, n (%)**			
Full- or part time job	27 (13.8%)	3 (4.1%)	0.06
Unemployed/retired	169 (85.7%)	71 (95.9%)	
**Income, n (%)**			
Low income	186 (94.9%)	72 (97.3%)	0.46
Medium income	6 (3.1%)	2 (2.7%)	
High income	4 (2.0%)	0 (0.0%)	
**Living place, n (%)**			
Urban	125 (63.8%)	51 (68.9%)	0.32
Mixed Urban-Rural	61 (31.1%)	17 (23.0%)	
Rural	10 (5.1%)	6 (8.1%)	
**Marital status, number, n (%)**			
Married/co-habiting	130 (66.3%)	36 (48.6%)	**0.01**
**Smoking Status, n (%)**			
Current	49 (25.0%)	18 (24.3%)	0.55
Never	15 (7.7%)	3 (4.1%)	
Former	132 (67.3%)	53 (71.6%)	
Stopped during intervention (reported at RCT follow-up)	3 (1.5%)	1 (1.4%)	0.09
**Pack years**	40.7 (22.4)	40.1 (18.5)	0.85
**COPD-SPECIFIC CHARARCTERISTICS - RCT baseline**			
**FEV1 % predicted**	53.4 (16.6)	46.3 (16.6)	0.002
**mMRC, mean (SD)**	1.9 (1.1)	2.6 (1.2)	<0.001
0, n (%)	10 (5.1%)	3 (4.1%)	<0.001
1	75 (38.3%)	10 (13.5%)	
2	60 (30.6%)	28 (37.8%)	
3	21 (10.7%)	7 (9.5%)	
4	30 (15.3%)	26 (35.1%)	
**GOLD classification, n (%)**			
Class 1	9 (4.6%)	0 (0.0%)	0.02
Class 2	102 (52.0%)	32 (43.2%)	
Class 3	67 (34.2%)	27 (36.5%)	
Class 4	16 (8.2%)	15 (20.3%)	
**Medication, COPD controller drugs, n (%)**			
None (0)	28 (14.3%)	7 (9.5%)	0.12
Usage of 1 type of medication	24 (12.2%)	9 (12.2%)	
Usage of 2 types of medication	77 (39.3%)	24 (32.4%)	

Usage of 3 types of medication	64 (32.7%)	29 (39.2%)	
Usage of 4 types of medication	3 (1.5%)	5 (6.8%)	
**RCT PERFORMANCE**			
**St George's Respiratory Questionnaire (SGRQ), Total score**			
RCT Baseline	44.1 (17.2)	48.3 (15.7)	0.07
RCT change (baseline to follow-up)	-3.0 (10.9)	-3.7 (9.0)	0.71
RCT MID (≥4 units) achieved, Yes, n (%)	66 (44.3%)	20 (44.4%)	0.99
**Six Minute Walk Test Distance (6MWD)**			
RCT Baseline	402.6 (95.7)	327.6 (100.5)	<0.001
RCT change (baseline to follow-up)	20.9 (36.7)	14.5 (49.5)	0.36
6MWD MID (≤30 m) achieved, Yes, n (%)	50 (35.0%)	12 (28.6%)	0.44
**HADS, mean (SD)**			
Anxiety score, baseline	5.0 (3.8)	4.6 (3.6)	0.49
Depression score, baseline	4.9 (3.8)	4.5 (3.4)	0.62
**Adherence to the intervention, n (%)**			
0-49%	43 (21.9%)	23 (31.1%)	0.27
50-74%	35 (17.9%)	10 (13.5%)	
75-100%	118 (60.2%)	41 (55.4%)	

Supplementary 3 Table text: Data are presented as mean ±SD or number (%). BMI: Body Mass Index. SGRQ Total Score: St George's Respiratory Questionnaire. HADS: Hospital Anxiety and Depression Scale; Sub-scores: symptoms of anxiety (HADS-A) and depression (HADS-D). mMRC: modified Medical Research Council dyspnoea score. Differences between-groups were tested using Student's t-test (two-tailed), paired-samples t-test, Chi^2, or Fischer's exact test. Statistical analyses were performed using statistical software STATA 18 (StataCorp LLC, Texas, USA). Statistical significance was reached at $p<0.05$.
